# Investigation of Basic Assumption for Contact Between Spheric Asperities in Rough Surface

**DOI:** 10.3390/ma18010150

**Published:** 2025-01-02

**Authors:** Caixia Guo, Feng Gao, Yangsen Mu, Yuewen Su, Canjun Wang, Xubo Li

**Affiliations:** 1 College of Mechanical Engineering, Baoji University of Arts and Sciences, Baoji 721016, China; 2 Shaanxi Key Laboratory of Advanced Manufacturing and Evaluation of Robot Key Components, Baoji 721016, China; 3 Shaanxi University Youth Innovation Team for Advanced Manufacturing and Testing of Key Robot Components, Baoji 721016, China

**Keywords:** contact model, finite element analysis, elliptical asperity, rough surfaces

## Abstract

Accurate analyses of contact problems for rough surfaces are important but complicated. Some assumptions, namely that all asperities can be approximated by a hemisphere with the same radius and assuming a Gaussian distribution of the asperity heights, are convenient but may lead to less accurate results. The purpose of this work is to investigate these assumptions and analyze the conditions under which they are valid. The finite element method is used to construct spherical asperity contact models with different radii and materials. The validity of the assumptions is assessed by comparatively analyzing the results of four models in terms of contact loads, contact radii, and average contact pressures under different yield strengths. The results show that these assumptions are fully applicable under elastic deformation. For plastic cases, the lower yield strength of the two contacting bodies is the dominant factor affecting the contact results. Assuming the same lower yield strength, the ratio of the yield strengths of two spheres has an influence on contact characteristics in the range from 1.2 to 3, but a negligible influence when the ratio is greater than 3. With an equivalent yield strength and yield ratio, the plastic contact of asperities can be analyzed in detail, which be conducive to clarifying the application scope of the above assumption. The work reported in this study provides some theoretical basis for an accurate contact model of rough surfaces.

## 1. Introduction

Engineering surfaces typically refers to surfaces used in engineering applications that are designed and manufactured to meet specific functional or performance requirements. Most engineering surfaces are rough, exhibiting microprotrusions or high points, commonly referred to as asperities. These microscopic irregularity significantly impact the contact behavior between surfaces such as friction, lubrication, wear, sealing, and electrical conductivity [1]. The investigation of contact problem requires the construction of a rough surface morphology [2]. Currently, rough surfaces are constructed using two main methods: statistical modeling and fractal modeling [3].

In non-lubricated or boundary lubrication cases, the contact between two rough surfaces actually takes place on a small number of asperities, and the real contact area accounts for only a small portion of the nominal contact area. Also, the contact and interaction of the asperities are the main cause of friction and wear, which are essentially related to the real surface morphology. So, the Coulomb friction theory and Archad wear model based on the macroscopic scale will no longer be applicable. A microscopic contact model was firstly proposed by Greenwood and Williamson [4] (G-W model), which successfully established the elastic contact model between asperities using the Hertzian contact theory and extended it from a single pair of asperities to the whole rough contact surface by a statistical method. Three kinds of basic assumptions are made in the G-W model. First, the asperities’ distribution is assumed to be isotropic. Second, the tops of the asperities are assumed to be semi-spheres with a uniform radius, but their heights vary randomly. Third, it is assumed that there is no merging of the asperities after being pressurized. However, the G-W model only considers the elastic deformation of the asperity without including the plasticity. Chang et al. [5] summarized earlier works to extend the G-W model and built a plastic contact model with volume conservation of an elastic–plastic hemisphere. Due to the simplification, the elastic–plastic contact area is expressed mathematically as a linear function only including a limiting elastic case and a full plasticity model. Zhao et al. [6] proposed a contact model for rough surfaces including three deformation states, namely elastic, elastoplastic, and plastic, and the smoothing transition of these different states at the yield point occurs for the contact pressure and bearing area of the asperities [7]. Jamari [8] carried out an experimental investigation to evaluate the models in earlier works for the contact of a deformable copper or aluminum sphere against a hard SiC ceramic flat in a fully plastic contact situation. Ghaednia et al. [1] summarized the latest models of single-hemispherical/elliptical elastoplastic contact and integrated them into a surface contact model framework by the stochastic method.

Numerical approaches, namely finite element analysis (FEA) [9,10] or the boundary element method (BEM) [11,12], have been used to calculate the contact pressure, contact stresses, and contact area of single asperity or rough surface contact problems. Hardy et al. [13] used a finite element analysis (FEA) to investigate the contact problem of a rigid sphere against an elastic–perfectly plastic half-space, and their predicted contact radii were in excellent agreement with the experimental results. Kral et al. [14] utilized FEA to study the repeated elastoplastic indentation of a half-space by a rigid spherical indenter with and without considering material strain hardening. Kogut and Etsion [15,16] performed a contact analysis of a single asperity subjected to normal force by the finite element method and defined the relationship between the deformations and loads for all three contact states, elastic, elastoplastic, and perfectly plastic, which also was extended to the rough surface contact using the statistical theory. These numerical approaches have been widely applied to solve rough surface contact problems under the necessary basic assumptions from the G-W model.

As mentioned above, three main hypothetical approximations in the G-W model are used to simplify the contact problem, which also face many problems in practical application. For instance, the assumption of the uniform radius for all asperities on the rough surface is not consistent with reality. Moreover, the measurement of the spherical asperity radius is also affected by the resolution of the instrument [17,18,19]. In the G-W model, plastic contact behavior cannot be properly captured when using roughness simplification.

Surface characteristics obtained based on statistics are limited by the resolution of the instrumentation, as well as the shortcomings of the statistical model itself, which do not accurately reflect the nature of surface roughness. So Majumdar and Bhushan [20,21] (M-B model) took advantage of the fact that fractal geometry can be used to characterize rough surfaces at multi-scales and replaced the statistical parameters with fractal parameters to establish a microscopic contact model for two-dimensional rough surfaces. Yan and Komvopoulos [22] extended the microcontact model of two-dimensional rough surfaces that satisfy the Weierstrass–Mandelbrot (W-M) function to three-dimensional rough surfaces that can describe the rough surface morphology more accurately, but their contact model is only involved in elastic–perfectly plastic conditions, ignoring the elastic–plastic state. In 1994, Wang and Komvopoulos [23,24] considered the role of the expansion factor of the microcontact size distribution and modified the M-B model. In Refs [18,25], an inverse relationship between the contact area and normal deformation for a single asperity was derived based on the modified M-B model, which is consistent with the deformation pattern of the M-B model.

Different from the statistical model, whose main parameters vary with the scale, the fractal surface model is scale-independent. However, the fractal model still adopts assumptions, i.e., the tip of the asperity is spherical, and the contact between the two rough surfaces is simplified as one between rigid smooth planes and the equivalent rough surfaces [26,27]. Similar to the statistical method, there is no theoretical basis for whether the two spheres can be equivalent to a rigid plane and for equivalent spherical contact under plastic deformation for fractal contact analysis [28].

The work reported in this paper aims at investigating some assumptions of the G-W model and addressing the questions mentioned above, which are (1) whether all asperities can be totally considered as spheres with the same radius for rough surface contact and (2) under what conditions does this equivalence hold. For this purpose, we built different contact models using FEA to analyze the contact behavior of spheres with different radii and material parameters. Data from the FEA model can be provided as a theoretical basis for the establishment of a more accurate rough surface contact model.

## 2. Finite Element Analysis Models and Validation

### 2.1. Problem Definition

According to the G-W model, the contact of two asperities can be represented by an equivalent sphere and a smooth rigid plane, and the radius R of the equivalent sphere can be defined as [7]

(1) $$\frac{1}{R}=\frac{1}{R_{1}}+\frac{1}{R_{2}}$$

where *R*_1_ and *R*_2_ are the radii of the two spheres, respectively. The equivalent modulus *E* of elasticity is defined as

(2) $$\frac{1}{E}=\frac{1-\nu_{1}^{2}}{E_{1}}+\frac{1-\nu_{2}^{2}}{E_{2}}$$

where *ν_i_*, *E_i_* are the Poisson’s ratio and elastic modulus of ball *i* (*i* = 1, 2), respectively.

**Figure 1:**
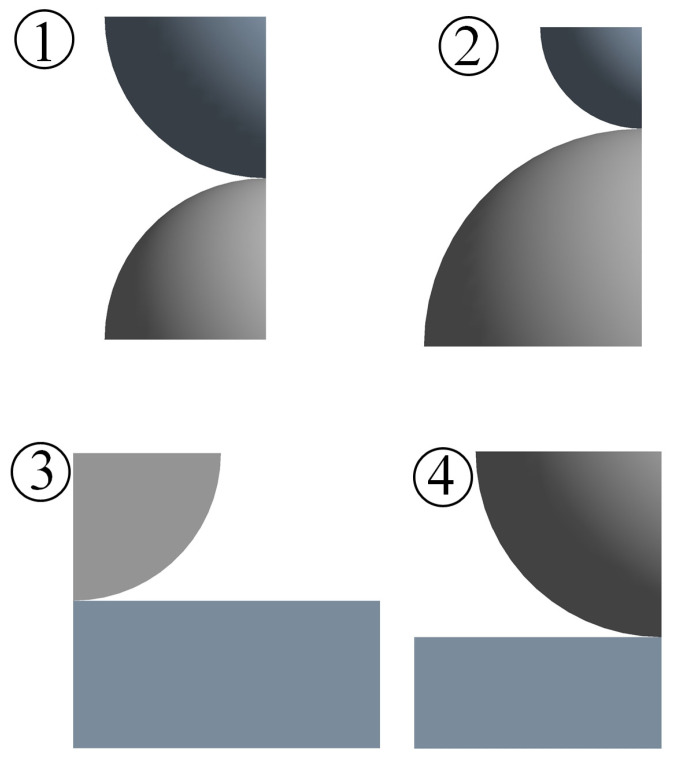
Contact configurations.

In order to analyze the above equivalence relations in detail, especially for the plastic case, the FEA software ANSYS Workbench 2023R1 (ANSYS 2023 R1) is used to construct the four models shown in Figure 1. The model assumes isotropic material properties. The four models correspond to the same equivalent model, i.e., the equivalent radius *R* is 50 mm as shown in Table 1; the equivalent elastic modulus *E* is 115.38 MPa. Since these models are mechanically axisymmetric, they can be modeled with 2D solid axisymmetric elements (Plane183 elements) to simplify the calculation. The meshing is performed using quadratic elements, with the finest mesh at the contact surface and coarser mesh away from the contact region. Each quadratic element has eight nodes attached to it. The number of elements is about 96,203 to 105,549, and the number of nodes is approximately 70,000, give or take. The contact surface of the rigid body is designated as the master surface, while the contact surface of the asperity body is specified as the slave surface. The assumptions of the contact condition include no friction and the absence of adhesion. The augmented Lagrange method is used as the contact algorithm. Calculations are performed using the Intel (R) Xeon (R) Gold 6238R CPU @ 2.20GHz 2.19 GHz model server from (Intel Corporation, Santa Clara, CA, USA).

### 2.2. Validation of Finite Element Model

**Figure 2:**
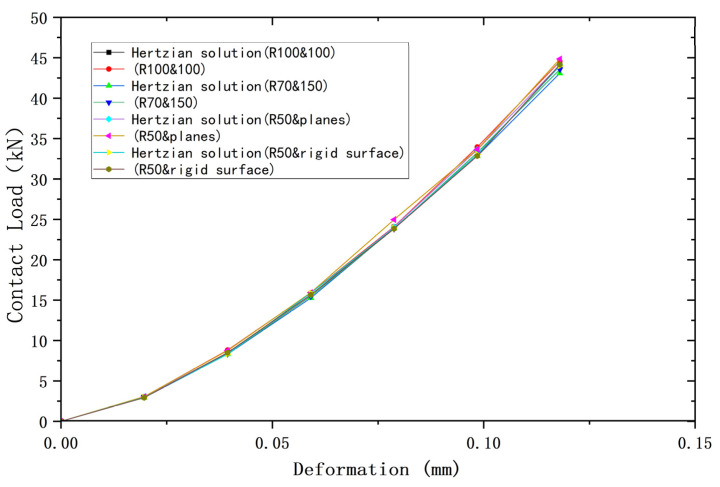
Deformation versus contact load.

**Figure 3:**
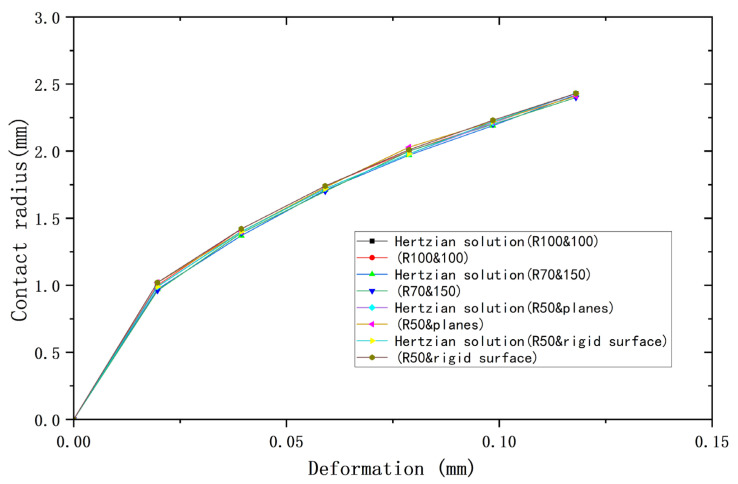
Deformation versus contact radius.

**Figure 4:**
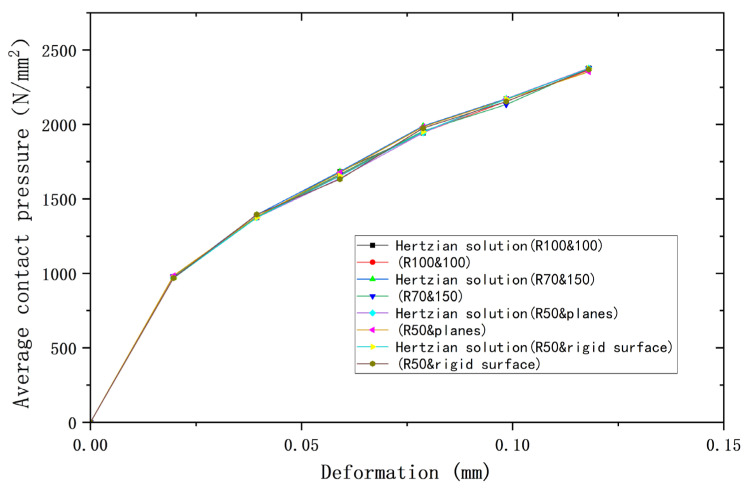
Deformation versus mean contact pressure.

Figure 2, Figure 3 and Figure 4 compare the results of the deformations obtained from the above four models and their corresponding Hertz theories with the contact load, contact radius, and average contact pressure, respectively, in the elastic range. It can be seen that the results of the four models are very close to the Hertz theory solutions. Table 2, Table 3 and Table 4 list the comparisons of the results of the four models when the deformation ω = 0.118 mm, and the maximum error in comparison with the Hertz solution is 1.7%, and the error is less than 1% in most cases, so the FEA results are sufficiently accurate.

**Figure 5:**
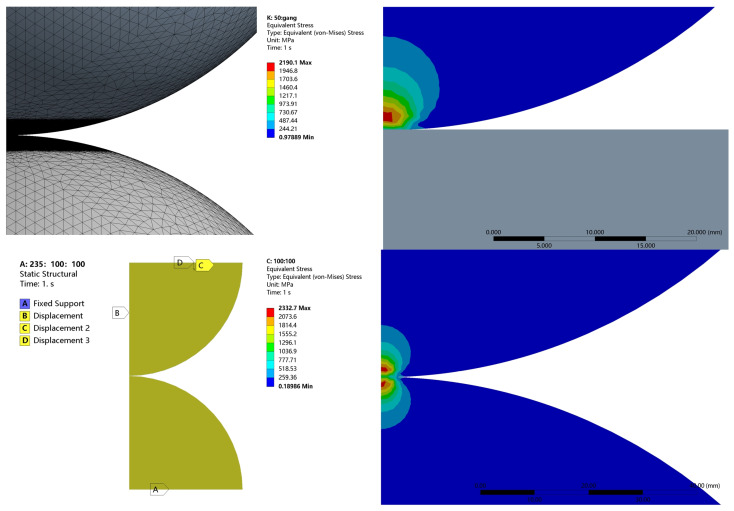
Contact stress distribution.

Figure 5 shows the von Mises stress distribution, where it can be seen that there is no stress in the rigid body, while the stress distribution in the spheres is roughly in line with the Hertz solution. The model was discretized using a fine mesh in the contact region to capture the stress variations accurately, and appropriate boundary conditions were applied, including fixed supports on the rigid body and a prescribed displacement at the interface between the sphere and rigid body to simulate realistic contact behavior. The maximum stress occurs below the contact surface and decreases outwards. The comparison of the errors and the distribution of the stresses also shows the reliability of the finite element results.

Meanwhile, the above results also prove that in the elastic range, the contact behavior of the four models with the same equivalent properties is very consistent with the Hertz solution. Therefore, the above equivalence is valid under elastic conditions.

## 3. Influence of Plasticity on Asperity Contact

In order to investigate whether the equivalence relationship in the G-W model holds under plastic conditions, the contact problem of asperities with plastic deformation is analyzed, and the material is set to be elastic–perfectly plastic, so as to compare and analyze the contact behavior under different yield strengths.

### 3.1. Cases for Asperities with Same Yield Strength

**Figure 6:**
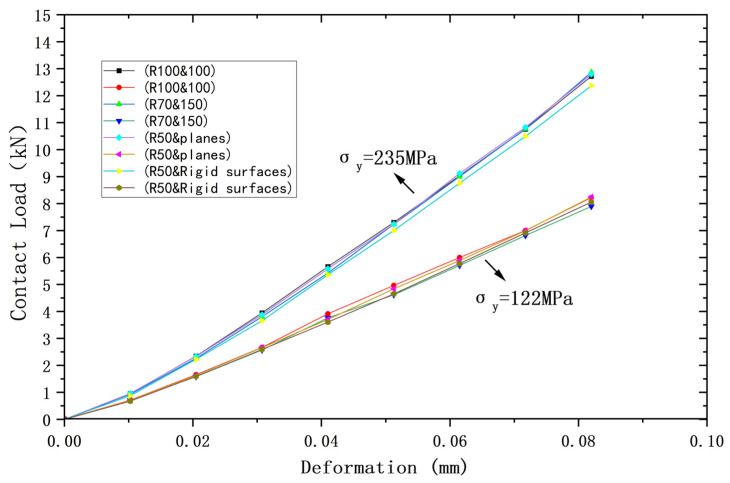
Deformation versus contact load for contacting bodies with the same yield strength.

**Figure 7:**
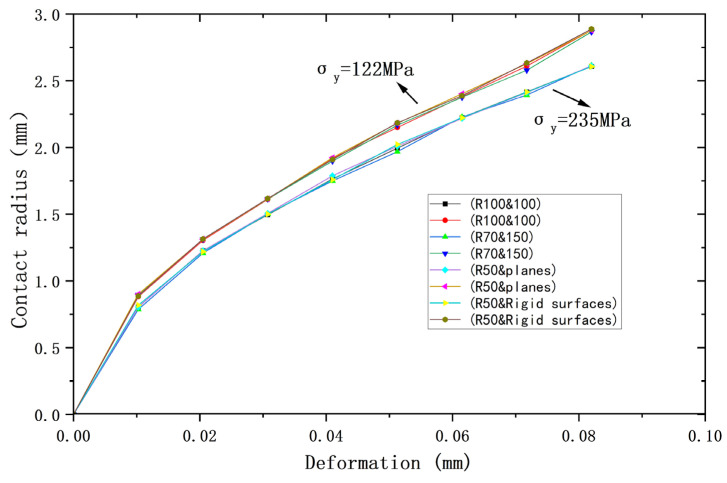
Deformation versus contact radius for contacting bodies with the same yield strength.

**Figure 8:**
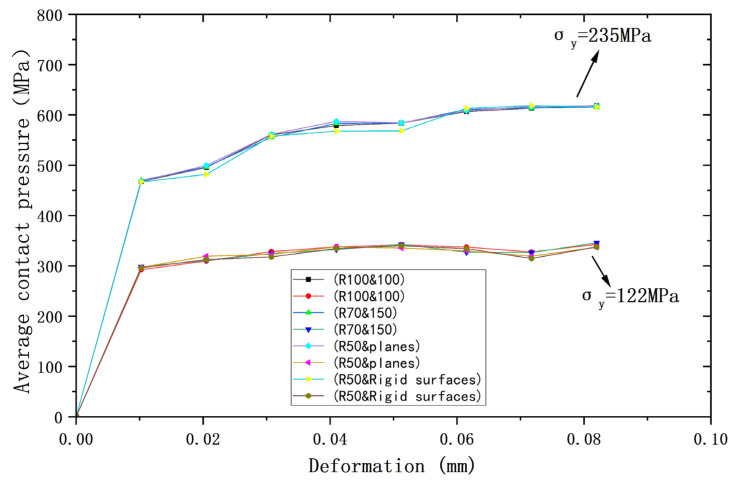
Deformation versus average contact pressure for contacting bodies with the same yield strength.

The yield strengths of the two contacting spheres are set as equal to compare and analyze their contact behavior under plastic conditions. Two yield strengths *σ*_y_ are used, namely *σ*_y_ = 235 MPa or 117.5 MPa. In particular, the yield strength of the rigid body is made to be +∞. Figure 6, Figure 7 and Figure 8 show the relationship between deformation and contact load, contact radius, and average contact pressure for the above four models, respectively. As can be seen from the figures, all contact behaviors for the two yield strengths are clearly separated into two curves, indicating that the results for the four models are nearly identical for the same yield strength.

For the same amount of deformation, the higher yield strength implies the larger contact load. Table 5 shows the contact loads of the four models for a deformation value of 0.082 mm, where the maximum difference between the four results is 3.9% for *σ*_y_ = 235 MPa and 4.2% for *σ*_y_ = 117.5 MPa, which represents nearly the same results for the four models at the same yield strength. Similar findings can be obtained for the contact radius and the average contact pressure, and the maximum deviations for the two yield strengths at a deformation value of 0.082 mm correspond to 0.73% and 2.6%, respectively, which represent nearly the same results at each yield strength. Table 6 and Table 7 show the contact radius and average contact pressure for the four models at a deformation value of 0.082 mm, which also lead to the same conclusion.

In summary, it is reasonable that the contact model of two rough surfaces can be represented as the contact of a rough surface with a smooth plane if the contacting bodies have the same yield strength.

### 3.2. Cases for Asperities with Same Yield Strength but Different Equivalent Yield Strength

The above analysis shows the yield strength plays an important role in the contact problem when considering plasticity. Therefore, using an analogous method to create the equivalent radius *R* and the equivalent modulus of elasticity *E*, the equivalent yield strength is defined as [29,30]

(3) $$\frac{1}{{\bar{\sigma }}_{y}}=\frac{1}{\sigma_{y1}}+\frac{1}{\sigma_{y2}}$$

where σ_yi_ is the yield strength of ball *i* (*i* = 1, 2).

**Figure 9:**
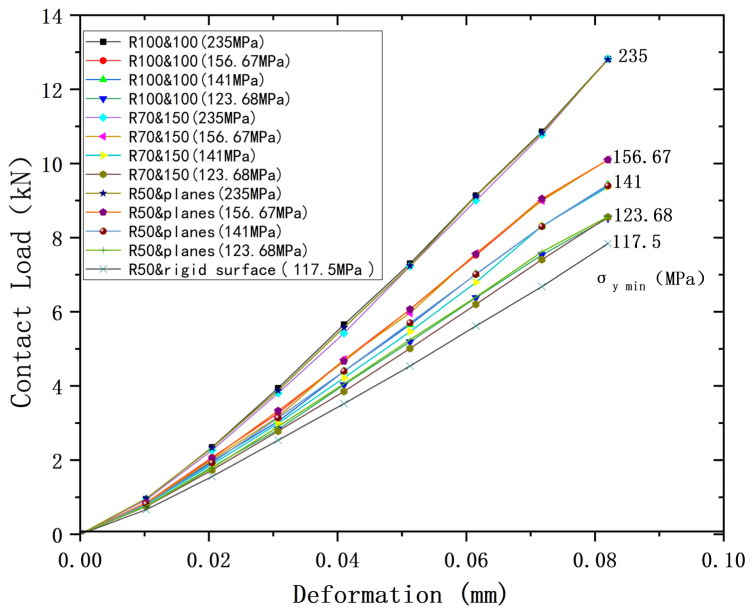
Deformation versus contact load for contacting bodies with different yield strengths but same equivalent yield strengths.

**Figure 10:**
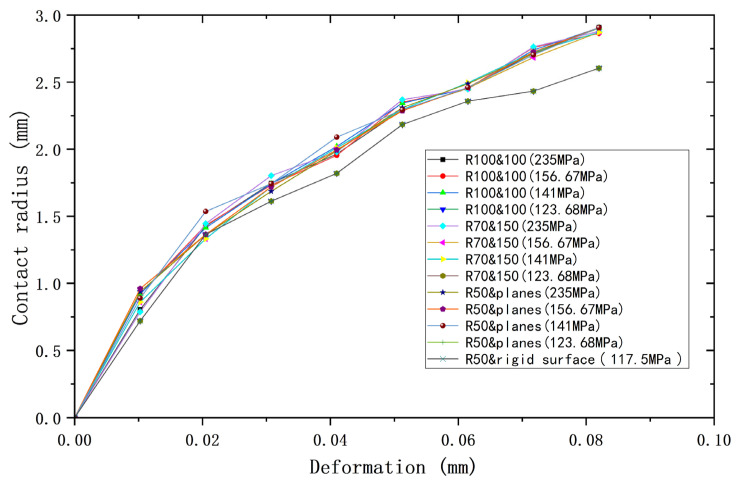
Deformation versus contact radius for contacting bodies with different yield strengths but same equivalent yield strengths.

**Figure 11:**
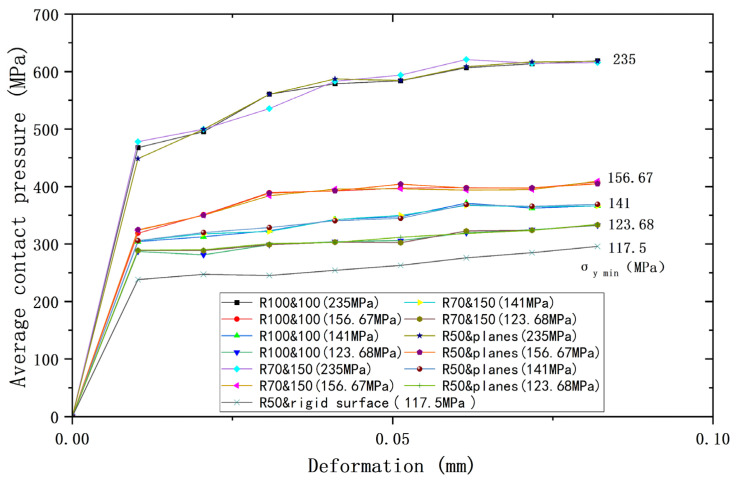
Deformation versus average contact pressure for contacting bodies with different yield strengths but same equivalent yield strengths.

**Figure 12:**
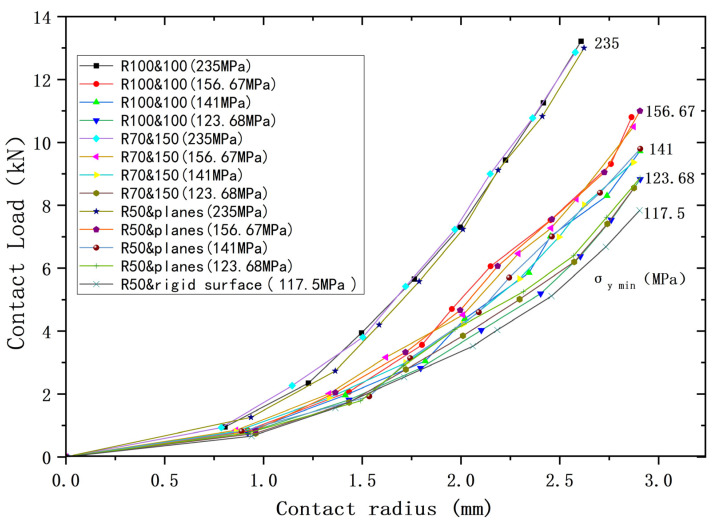
Load versus contact radius for contacting bodies with different yield strengths but same equivalent yield strengths.

Since models ③ and ④ are both of the contact between a sphere with plane, they can be considered as one model. So, three cases can be used for analysis, plus ① and ②, which can then be combined with the yield strength parameters (shown in Table 8) to form a total of 13 cases. Figure 9, Figure 10 and Figure 11 show the deformation versus contact load, contact radius, and average contact pressure for these 13 cases. Although these models have the same equivalent radius, equivalent modulus of elasticity, and equivalent yield strength, their contact behavior is not identical. As the deformation increases, contact loads and average contact pressures change significantly differently and can be roughly categorized into two groups. The lower yield strengths (σ_y2_ = 156.67 MPa, 141 MPa, 123.68 MPa) are categorized into one group, while *σ*_y2_ = 235 MPa is the other group. However, the difference in contact radius with deformation for the above 13 cases is less. Figure 12 shows the relationship between contact radius and contact load, with a similar situation of division into two groups.

It can be seen that the above contact behavior is closely related to the lower yield strength *σ*_y2_. The analysis results of the contact models with the same relative lower yield strength are more approximate, indicating that the contact behavior under plastic conditions is dominated by the lower yield strength σ_y2_.

### 3.3. Cases for Asperities with Same Lower Yield Strength but Different Ratio

From the above, it is clear that the lower yield strength *σ*_y2_ has an important effect on contact behavior under plastic conditions. Therefore, instead of equivalent yield strength, the yield strength ratio *K* is used, i.e., the lower yield strength *σ*_y2_ is set to be the same in different models, but σ_y1_ = *Kσ*_y2_. The values of *K* are shown in Table 9.

**Figure 13:**
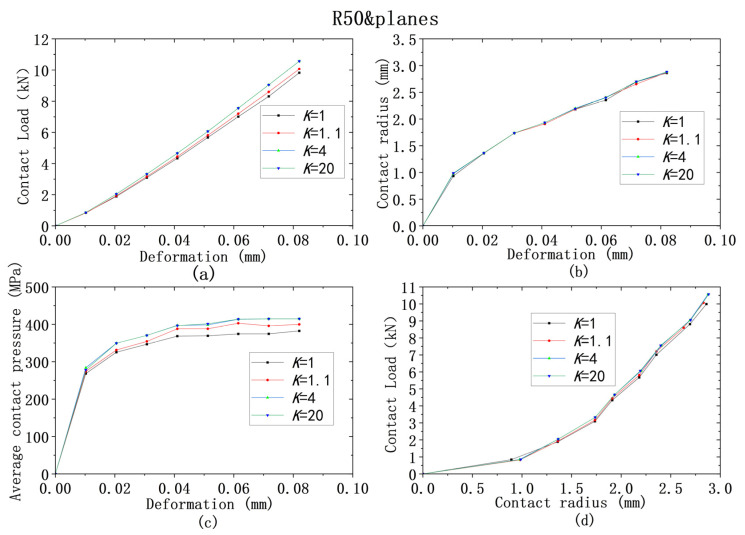
Contact analysis results for cases with same lower yield strengths but different ratios (R50&planes): (**a**–**c**) for deformation versus contact load, contact radius, and average contact pressure; (**d**) for contact radius versus contact load.

**Figure 14:**
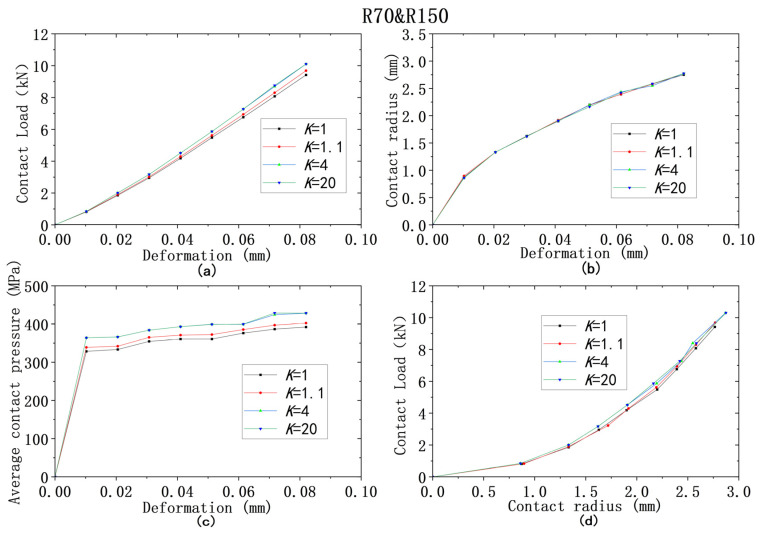
Contact analysis results for cases with same lower yield strengths but different ratios (R70&R150): (**a**–**c**) for deformation versus contact load, contact radius, and average contact pressure; (**d**) for contact radius versus contact load.

**Figure 15:**
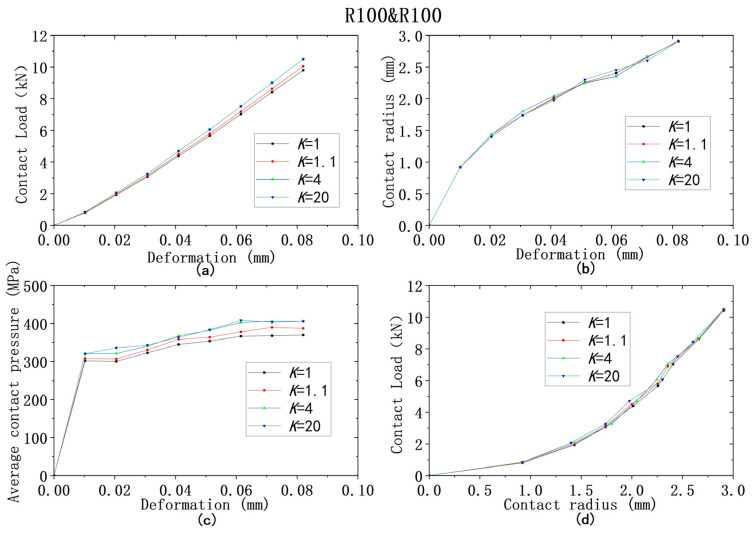
Contact analysis results for cases with same lower yield strengths but different ratios (R100&R100): (**a**–**c**) for deformation versus contact load, contact radius, and average contact pressure; (**d**) for contact radius versus contact load.

**Figure 16:**
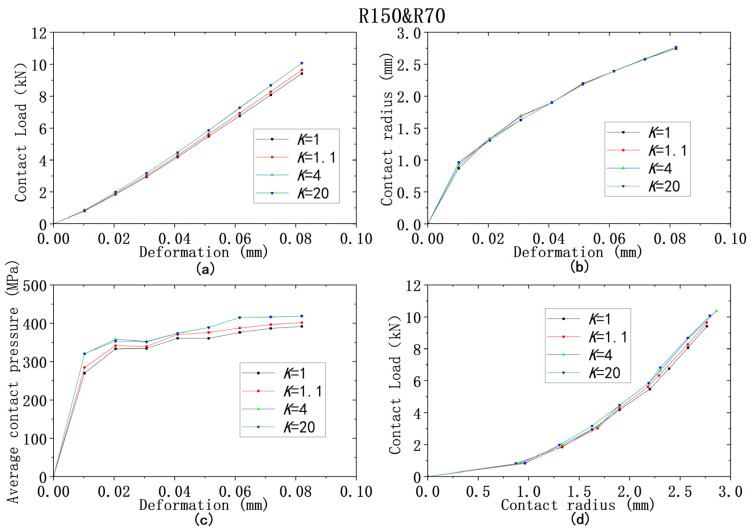
Contact analysis results for cases with same lower yield strengths but different ratios (R150&R70): (**a**–**c**) for deformation versus contact load, contact radius, and average contact pressure; (**d**) for contact radius versus contact load.

**Figure 17:**
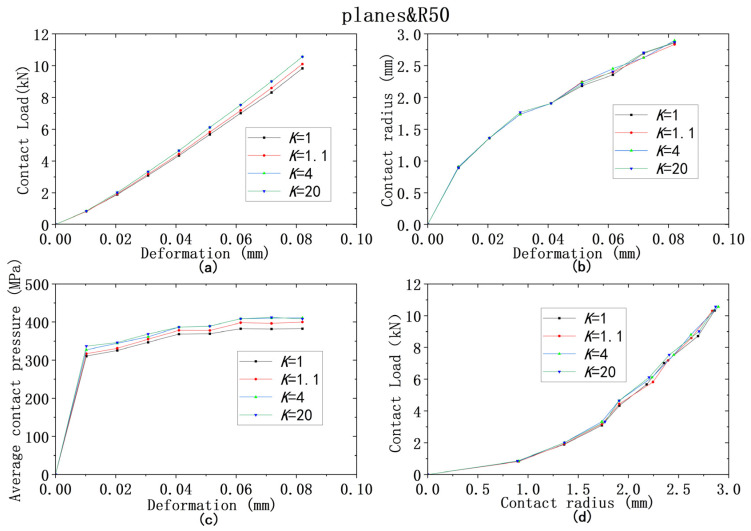
Contact analysis results for cases with same lower yield strengths but different ratios (plane&R50): (**a**–**c**) for deformation versus contact load, contact radius, and average contact pressure; (**d**) for contact radius versus contact load.

For all four models from ① to ④, the yield strength combinations from Table 9 are used for analysis of contact behaviors. Figure 13, Figure 14, Figure 15, Figure 16 and Figure 17 show that the results of deformation vs. contact load, deformation vs. contact radius, and deformation vs. average contact pressure have the same trend, which are reflected by three lines in Figures. Among them, the results from the cases using the larger yield ratios (yield ratio of 4, 20) are so close that they almost overlap. It can be reasonably inferred that most contact behaviors will no longer change when *K* is greater than a critical value, which is between 1.1 and 4. However, for the relationship between contact radius and load, the results are very close for all four yield strength ratios.

### 3.4. Cases for Asperities with Same Lower Yield Strength and Ratio K

**Figure 18:**
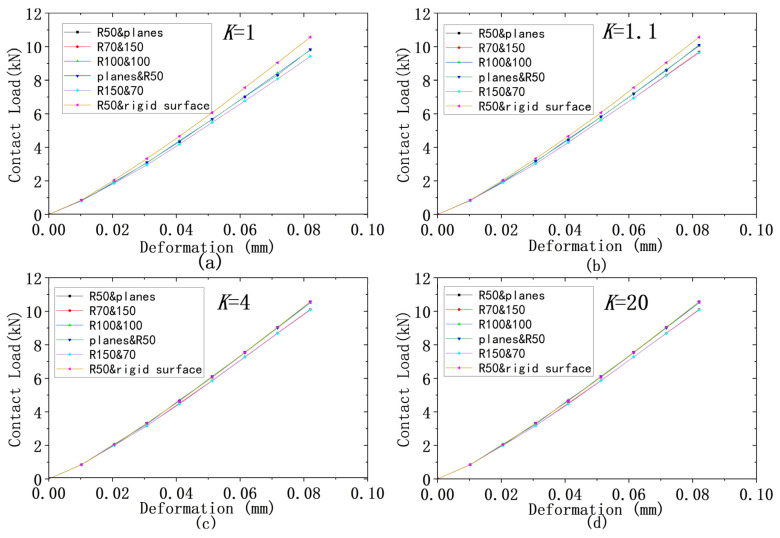
Deformation versus contact load for different models at different *K*s: (**a**–**d**) for *K* = 1, 1.1, 4, 20.

In the following, the contact problems will be analyzed when the lower yield strength σ_y1_ and yield strength ratio *K* are both the same. Figure 18 shows that the relationship between deformation and contact loading for all different geometrical parameters are basically close, despite using a different yield strength ratio *K*. The maximum difference between the contact loads obtained for different *K*s is 6.4% at a deformation of 0.051 mm and 9.1% at a deformation of 0.082 mm. The difference enlarges with the increase in deformation. So, if the contact load is to be predicted from the deformation, it is necessary to take the influence of yield strengthen ratios into account.

**Figure 19:**
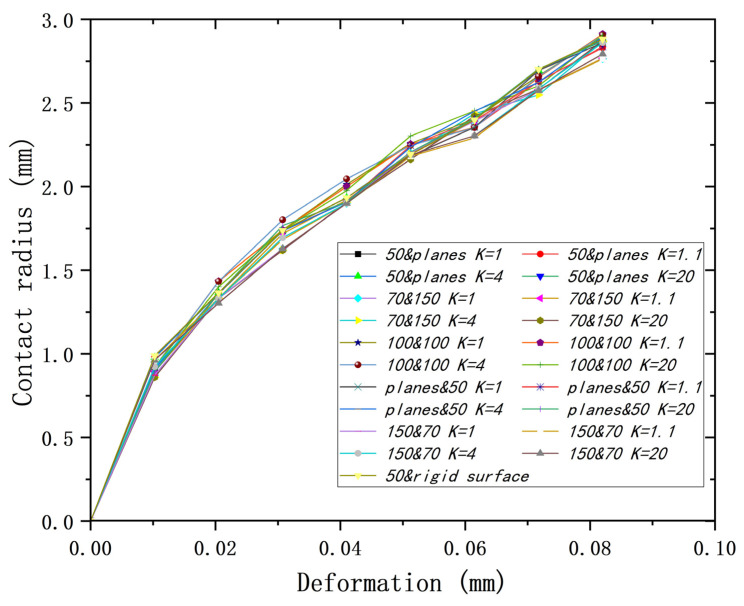
Deformation versus contact radius for different models at different *K*s.

Figure 19 shows the curves of deformation to contact radiuses for all models with different *K*s. It can be seen that all the curves almost overlap (maximum error of 4.8% at a deformation of 0.082 mm), which indicates that the main factor affecting the deformation–contact radius relationship is the lower yield strength, which is independent of the yield strength ratio *K*.

**Figure 20:**
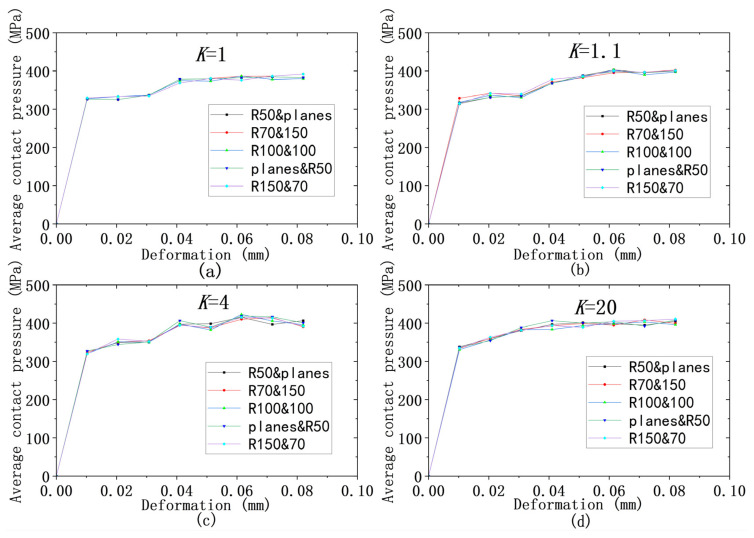
Deformation versus average contact pressure for different models at different *K*s: (**a**–**d**) for *K* = 1, 1.1, 4, 20.

**Figure 21:**
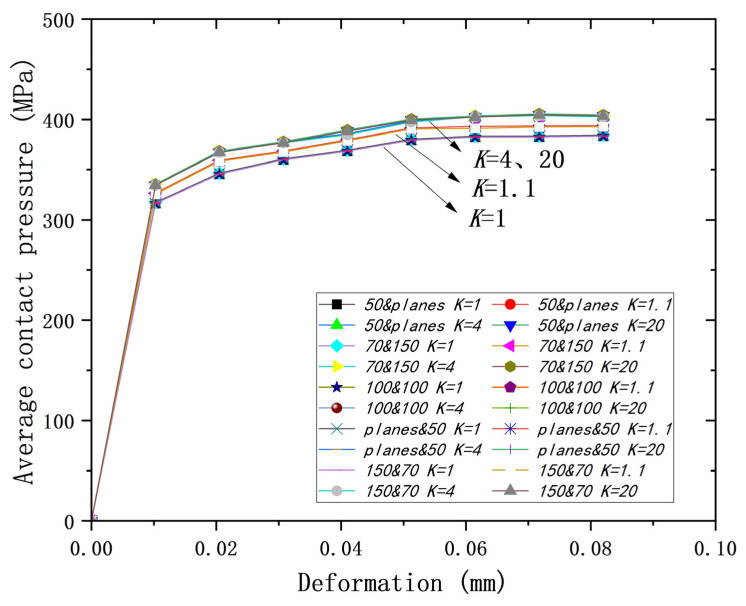
Deformation versus average contact pressure for all models at different *K*s.

As shown in Figure 20, a similar situation happens to the relationship of deformation to average contact pressure. It can be seen that the deformation–mean contact pressure curves obtained from different models are nearly the same. The curves in Figure 21 are roughly divided into three according to the different yield strength ratios, but the difference in the average contact pressure is not significant for the different yield ratios under the same deformation. When the deformation is 0.082 mm, the maximum difference in the average contact pressure is 5%, which is within the allowable range, and the maximum difference tends to be constant with the increase in deformation amount.

**Figure 22:**
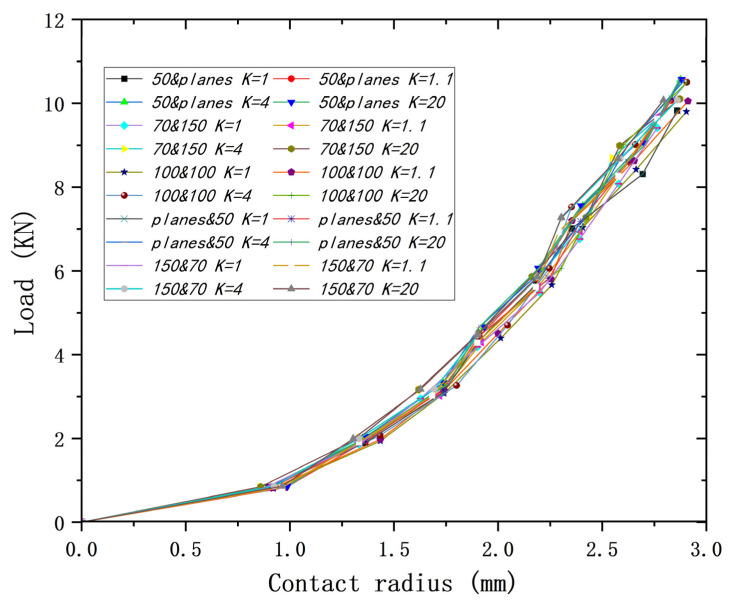
Contact radius versus load for different models at different *K*s.

Figure 22 shows the curves of contact radius–contact load for all different yield strength ratios *K* at the same lower yield strength. It can be found that the maximum difference is 9.3% for a contact radius of 1.91 mm and 5.4% for a contact radius of 2.86 mm for the same lower yield strength. In addition, as the contact radius increases, the difference between the maximum and minimum values of the load does not increase significantly, resulting in the difference decreasing. So, only with the same lower yield strength, the relationship between contact radius and load is very approximate for the different models.

## 4. Conclusions

This work investigated the common assumption that contact problems can be reduced to the one between a rigid surface and an equivalent rough surface by FEA and discussed the conditions under which it is established. The analytical findings reveal the following:

The assumptions are fully established under elastic conditions. If considering plasticity, the above simplification method only holds under the condition that the yield strengths of the two spheres should be equal.

However, contact behaviors are different for the case of two contacting bodies with different yield strengths, which is dominated by the lower yield strength of the two. It should be used as the key indicator for determining the validity of the assumptions. If the lower yield strengths are the same, the contact results are slightly different for the smaller ratio (the higher yield strength to the lower one). However, the difference is almost negligible when the ratio is greater than a critical yield ratio (between 1.2 and 3). If focusing only on the relationship between contact radius and load, the assumption is right only with the same lower yield strength. For a comprehensive contact analysis, the assumptions are sufficiently correct with the preconditions that both lower yield strength and ratio should be equal.

The finding in this work can be a basis for future studies, such as modeling more accurate contact behavior for rough surfaces with an advanced materials model, e.g., material hardening and nonlinear strain. This may lead to the creation of model sets for the friction, wear, and failure analysis of assembly joints in the automotive and aerospace sectors.

## Figures and Tables

**Table 1:** Case details of asperity contact configurations.

Contact Configuration	Model			
	①	②	③	④
*R*_1_ (mm)	100	70	50	50
*R*_2_ (mm)	100	150	+∞	+∞
*E*_1_ (GPa)	210	210	210	105
*E*_2_ (GPa)	210	210	210	+∞
*ν*_1_	0.3	0.3	0.3	0.3
*ν*_2_	0.3	0.3	0.3	

**Table 2:** Contact load (ω = 0.118 mm).

	Load/kN	Inaccuracies
Hertz solution	44.095	0
Model ①	44.556	1%
Hertz solution	43.081	0
Model ②	43.556	1.1%
Hertz solution	44.096	0
Model ③	44.844	1.7%
Hertz solution	43.995	0
Model ④	44.156	0.4%

**Table 3:** Contact radius (ω = 0.118 mm).

	Contact Radius/mm	Inaccuracies
Hertz solution	2.43	0
Model ①	2.43	0
Hertz solution	2.42	0
Model ②	2.4	0.8%
Hertz solution	2.43	0
Model ③	2.41	0.8%
Hertz solution	2.43	0
Model ④	2.43	0

**Table 4:** Average contact pressure (ω = 0.118 mm).

	Average Contact Pressure/MPa	Inaccuracies
Hertz solution	2379	0
Model ①	2365	0.6%
Hertz solution	2374	0
Model ②	2377	0.13%
Hertz solution	2379	0
Model ③	2354	1%
Hertz solution	2375	0
Model ④	2370	0.2%

**Table 5:** Load with deformation of 0.082 mm.

		Model	Model ①	Model ②	Model ③	Model ④
	Load (kN)					
Yield Strength						
*σ*_y_ = 235 MPa			12.7166	12.8632	12.7988	12.3668
*σ*_y_ = 122 MPa			8.2204	7.8944	8.2436	8.0552

**Table 6:** Contact radius with deformation of 0.082 mm.

		Model	Model ①	Model ②	Model ③	Model ④
	Contact					
Yield Strength Radius (mm)						
*σ*_y_ = 235 MPa			2.608	2.614	2.611	2.606
*σ*_y_ = 122 MPa			2.878	2.866	2.877	2.887

**Table 7:** Average contact pressure with deformation of 0.082 mm.

	Model	Model ①	Model ②	Model ③	Model ④
Yield Strength	Average Contact Presure (MPa)				
*σ*_y_ = 235 MPa		618.53	615.597	617.83	615.63
*σ*_y_ = 122 MPa		342.809	345.93	337.02	337.63

**Table 8:** Combinations of different equivalent yield strengths.

Parameters	Value				
*σ*_y1_/MPa	235	470	705	2350	117.5
*σ***_y2_**/MPa	235	156.67	141	123.68	+∞

**Table 9:** Combinations of different yield strength ratios K.

K	1	1.1	4	20	
*σ*_1_/MPa	156.67	156.67	156.67	156.67	156.67
*σ*_2_/MPa	156.67	172.337	626.68	3313.4	+∞

## Data Availability

The original contributions presented in the study are included in the article, further inquiries can be directed to the corresponding author.
